# Research topic: neurosteroids

**DOI:** 10.3389/fendo.2012.00126

**Published:** 2012-10-29

**Authors:** Hubert Vaudry, Kazuyoshi Tsutsui

**Affiliations:** ^1^University of RouenRouen, France; ^2^Waseda UniversityTokyo, Japan

Thirty years ago, the group of Baulieu and colleagues discovered that certain steroid hormones were present in higher amounts in the brain than in the plasma, and also found that suppression of circulating steroids by adrenalectomy and castration does not affect the concentration of pregnenolone, dehydroepiandrosterone, and their sulfate esters in the rat brain. These seminal observations led to the concept that the brain, in very much the same way as the adrenal cortex, testis, ovary, and placenta, is capable of synthesizing steroids. These brain born steroids, called neurosteroids, have been found to exert a vast array of biological activities. A number of steroidogenic enzymes have now been identified in the central nervous system by immunohistochemistry and *in situ* hybridization, and the neuronal and hormonal mechanisms regulating the biosynthesis of neurosteroids have been partially elucidated. The aim of this Research Topic is to celebrate three decades of research on neurosteroids by gathering a bouquet of review papers and original articles from leading scientists in the flourishing field of neurosteroids. We are deeply indebted to all the authors who have contributed to this Research Topic and to the dedicated reviewers who helped us reaching the highest quality standards. We gratefully acknowledge the valuable support of the Frontiers team and Mrs. Catherine Beau for her invaluable help in the processing of manuscripts.

